# Cost-Effectiveness Analysis of Myopia Progression Interventions in Children

**DOI:** 10.1001/jamanetworkopen.2023.40986

**Published:** 2023-11-02

**Authors:** Sylvia Agyekum, Poemen P. Chan, Prince E. Adjei, Yuzhou Zhang, Zhaohua Huo, Benjamin H. K. Yip, Patrick Ip, Ian C. K. Wong, Wei Zhang, Clement C. Tham, Li Jia Chen, Xiu Juan Zhang, Chi Pui Pang, Jason C. Yam

**Affiliations:** 1 Department of Ophthalmology and Visual Sciences, The Chinese University of Hong Kong, Hong Kong SAR, China; 2 Hong Kong Eye Hospital, Hong Kong SAR, China; 3 Lam Kin Chung. Jet King-Shing Ho Glaucoma Treatment and Research Centre, Department of Ophthalmology and Visual Sciences, The Chinese University of Hong Kong, Hong Kong SAR, China; 4 Department of Ophthalmology and Visual Sciences, The Prince of Wales Hospital, Hong Kong SAR, China; 5 Joint Shantou International Eye Center of Shantou University and the Chinese University of Hong Kong, Shantou, China; 6 School of Life Science, Department of Biomedical Engineering, University of Electronic Science and Technology, Chengdu, China; 7 Department of Computer Engineering, Kwame Nkrumah University of Science and Technology, Kumasi, Ghana; 8 Jockey Club School of Public Health and Primary Care, The Chinese University of Hong Kong, Hong Kong SAR, China; 9 Department of Pediatrics and Adolescent Medicine, Li Ka Shing Faculty of Medicine, The University of Hong Kong, Hong Kong SAR, China; 10 Centre for Safe Medication Practice and Research, Department of Pharmacology and Pharmacy, Li Ka Shing Faculty of Medicine, The University of Hong Kong, Hong Kong SAR, China; 11 Tianjin Eye Hospital, Tianjin Key Lab of Ophthalmology and Visual Science, Nankai University Affiliated Eye Hospital, Clinical College of Ophthalmology Tianjin Medical University, Tianjin, China; 12 Department of Ophthalmology, Hong Kong Children Hospital, Hong Kong SAR. China; 13 Hong Kong Hub of Pediatric Excellence, The Chinese University of Hong Kong, Hong Kong SAR, China

## Abstract

**Importance:**

Several interventions exist for treating myopia progression in children. While these interventions’ efficacy has been studied, their cost-effectiveness remains unknown and has not been compared.

**Objective:**

To determine cost-effective options for controlling myopia progression in children.

**Design, Setting, and Participants:**

In this cost-effectiveness analysis, a Markov model was designed to compare the cost-effectiveness of interventions for controlling myopia progression over 5 years from a societal perspective in a simulated hypothetical cohort of patients aged 10 years with myopia. Myopia interventions considered included atropine eye drops, 0.05% and 0.01%, defocus incorporated multiple segment spectacles, outdoor activity, soft contact lenses (daily disposable and multifocal), rigid gas-permeable contact lenses, progressive addition lenses, bifocal spectacle lenses, orthokeratology, highly aspherical lenslets (HALs), and red light therapy; all interventions were compared with single-vision lenses. Deterministic and probabilistic sensitivity analysis determined the association of model uncertainties with the cost-effectiveness. Costs were obtained from the charges of the Hospital Authority of Hong Kong and The Chinese University of Hong Kong Eye Center.

**Main Outcome and Measures:**

The mean costs (in US dollars) per child included the cost of hospital visits, medications, and optical lenses. The outcomes of effectiveness were the annual spherical equivalent refraction (SER) and axial length (AL) reductions. Incremental cost-effectiveness ratios (ICERs) were calculated for each strategy relative to single-vision lenses over a time horizon of 5 years.

**Results:**

Outdoor activity, atropine (0.05%), red light therapy, HALs, and orthokeratology were cost-effective. The ICER of atropine, 0.05%, was US $220/SER reduction; red light therapy, US $846/SER reduction; and HALs, US $448/SER reduction. Outdoor activity yielded a savings of US $5/SER reduction and US $8/AL reduction. Orthokeratology resulted in an ICER of US $2376/AL reduction.

**Conclusions and Relevance:**

These findings suggest that atropine eye drops, 0.05%, and outdoor activity are cost-effective for controlling myopia progression in children. Though more expensive, red light therapy, HALs, and orthokeratology may also be cost-effective. The use of these interventions may help to control myopia in a cost-effective way.

## Key Points

**Question:**

What is the most cost-effective strategy for controlling myopia in children?

**Findings:**

This cost-effectiveness analysis compared 13 myopia progression interventions for children using a Markov model. Over 5 years, atropine, 0.05%, and outdoor activity were cost-effective, with an incremental cost-effectiveness ratio of US $220 per spherical equivalent reduction for atropine, 0.05%, and a cost savings of US $5 per spherical equivalent reduction for outdoor activity; red light therapy, highly aspherical lenslets, and orthokeratology could also be cost-effective, although at higher costs.

**Meaning:**

These results suggest that the use of certain interventions may help reduce myopia progression in children in a cost-effective way.

## Teaser

This economic evaluation study uses a Markov model to assess the cost-effectiveness of the current myopia progression interventions in a hypothetical cohort of children aged 10 years.

## Introduction

Myopia (nearsightedness) is a major cause of visual impairment. Worldwide, approximately 153 million individuals older than 5 years have visual impairment due to uncorrected refractive errors, and of these, 8 million are blind.^1^ It has been estimated that the prevalence of myopia will increase from 2.6 billion in the year 2020 to 4.8 billion by the year 2050 (49.8% of the world’s population).^2^ The high and rising prevalence of myopia has become a major global health concern because of the potential long-term complications, including cataracts, myopic macular degeneration, glaucoma, and retinal detachments.^3^ Myopia, in particular high myopia, substantially affects the social, educational, and economic aspects of life.^4^

The health cost associated with myopia is high mainly due to the direct costs of myopia correction. The estimated annual cost of myopia in US dollars was approximately $4 to $7 billion in the United States^5^ and $25 to $755 million in Singapore.^6,7^ The cost increases when myopia-related morbidities cause visual impairment and blindness. For instance, patients with myopic choroidal neovascularization incurred a direct medical cost of €1629 (US $1743) more than those without.^8^ In 2015, the global potential productivity loss due to uncorrected myopia and myopic macular degeneration in US dollars was $244 billion and $6 billion, respectively.^9^

The efficacy of different interventions to halt or slow myopia progression has been evaluated. These include medication eye drops (atropine, pirenzepine, cyclopentolate, and timolol),^10,11,12,13,14,15^ spectacles (progressive addition lenses, prismatic bifocal spectacle lenses, peripheral defocus modifying spectacle lenses, and defocus incorporated multiple segments spectacles [DIMS]),^16,17,18^ and contact lenses (orthokeratology, peripheral defocus modifying contact lenses, rigid gas-permeable contact lenses [RGPCLs], and soft contact lenses).^19,20,21,22,23,24^ Higher doses of atropine were the most efficacious modality, while orthokeratology, pirenzepine, peripheral defocus modifying contact lenses, cyclopentolate, and prismatic bifocal spectacle lenses had moderate effects.^25^

However, the cost-effectiveness of these interventions requires a thorough evaluation.^26^ To date, only 1 study has examined the cost-effectiveness of photorefractive screening in 11-year-old children followed by treatment with atropine eye drops, 0.01%, for the children who have positive screen results for myopia.^27^ The knowledge gap on the cost-effectiveness of myopia interventions has to be addressed to provide pivotal data for health policy planning and maximize health outcomes under limited resources.^5,28^ A comprehensive cost-effectiveness analysis that examines a broad spectrum of myopia control interventions (ie, pharmacological, spectacles, and contact lenses) is required. Therefore, this study aimed to determine the cost-effectiveness of the current myopia progression interventions in 10-year-old children.

## Methods

### Study Design

We conducted a model-based economic evaluation on the cost-effectiveness of interventions to prevent myopia progression in children. We compared the effect of low-dose atropine eye drops (0.05% and 0.01%), DIMS, outdoor activity, soft contact lenses (daily disposable soft contact lenses [MiSight; CooperVision] and multifocal soft contact lenses [MSCLs]), RGPCLs, PALs, BSLs, orthokeratology, HALs, and red light therapy with single-vision lenses (SVLs) for controlling myopia progression over a 5-year period. Single-vision lenses were chosen as the comparator because this is the traditional approach to manage myopia. We set a 5-year period because previous clinical trials involving myopia interventions usually did not exceed this period.^29^ The target population consisted of a hypothetical cohort of children aged 10 years with myopia. Model inputs were obtained from published literature. Hence, the study was exempted from the need for review and informed consent by the research ethics board of The Chinese University of Hong Kong. Model parameters were varied within plausible ranges of values to determine their effect on the model in both deterministic and probabilistic sensitivity analyses. Cost-effectiveness acceptability curves were used to indicate the probability (proportion of iterations) that a strategy was cost-effective over a willingness-to-pay (WTP) threshold range of US $0 to $10 000 per spherical equivalent refraction (SER) reduction or axial length (AL) reduction. Minimum and maximum values of effectiveness were derived as the range of 95% CIs of myopia reduction.^30^ We reported the methods and results of this study in accordance with the Consolidated Health Economic Evaluation Reporting Standards (CHEERS) reporting guideline.

### Model Design and Myopia Progression

A transition-state model was implemented in TreeAge Pro software, version 2022 (TreeAge Pro Healthcare) to simulate the effect of prescribed interventions on myopia progression and to monitor the transition between health states. We used a Markov model with low, moderate, and high myopia as the health states (eFigure 1 in Supplement 1). Low myopia was defined as the spherical equivalent of −0.50 to −2.99 diopters (D), moderate myopia as −3.00 to −5.99 D, and high myopia as −6.0 D or greater.^31,32^ Transition probabilities were calculated based on annual myopia progression rate (eMethods in Supplement 1). The annual myopia progression rates were obtained from randomized clinical trials (eTable in Supplement 1) and were categorized as slow (<0.5 D), intermediate (0.5 to 0.99 D), and rapid (≥1.0 D) progression.^11,24,33^ In our model, the proportion of patients starting in each myopic state was based on the prevalence data reported by Yam et al,^34^ assuming that patients would progress from the less serious myopic state to the more serious state. The risk of progressing from one state to another could be reduced by treatment. We also assumed that myopia progression was irreversible. Hence, once a patient develops a more severe myopic state, the patient cannot revert to a lower myopic state. Additionally, we assumed that myopia progression was directly related to myopic state; patients with low and high myopia would have slow and rapid myopia progression, respectively.^35,36^ In our model, contact lenses or spectacles were changed when patients progressed to high myopia.

### Costs and Outcomes of Effectiveness

Analysis was performed from a societal perspective, including direct and indirect costs. Direct costs included costs of consultations, follow-up visits, optometric services (refraction), specialized ophthalmic services, spectacles, contact lens solutions, and medications. Indirect costs included costs of adverse events and caretakers’ loss of productivity (time spent and wages lost in accompanying a child for interventions). Adverse events with the use of atropine eye drops were allergic conjunctivitis and photophobia requiring photochromatic glasses.^11^ Adverse events with the use of contact lenses consisted of bacterial keratitis, cornea infiltrates, allergies, hordeolum, and corneal staining.^37,38^ We assumed that patients spent a maximum of 2 hours in the clinic. Loss of productivity was calculated based on the median annual earnings from the Census and Statistics Department of Hong Kong.^39^

Costs were determined based on the published charges of the Hospital Authority of Hong Kong,^40^ the Chinese University of Hong Kong Eye Centre, and key informants. Except for patients given orthokeratology, spectacle lenses (DIMS, PALs, HALs, and BSLs), or contact lenses, all patients were assumed to wear SVLs and would at least incur the cost of spectacles. Hence, the baseline cost of outdoor activity was the cost of spectacles. Given that outdoor activity should otherwise have no direct cost, we investigated how our model would be affected if the cost was zero and if the cost included productivity losses related to spending time outdoors in sensitivity analysis. All costs were based on item costs in the year 2022, collected in Hong Kong dollars (HK $) and converted to US dollars (US $) at a rate of HK $7.85 per US $1.^41^ All costs were discounted at an annual rate of 3% as recommended by the World Health Organization.^42^

The outcomes for effectiveness were defined in terms of the change in SER and AL over 1 year, determined from published meta-analysis from literature.^30,43^ The annual change in SER and AL for the untreated cohort was obtained from the placebo cohort of Yam et al.^11^ Table 1 shows the model parameters. The main outcome of our study was the incremental cost-effectiveness ratio (ICER), calculated as (TCa − TCb)/(Ea − Eb), where TC represents the total costs, E is the effectiveness gain at the end of the calculation period, a is the treatment paradigm of interest, and b is the comparator.

**Table 1.** Model Parameters and Range for Sensitivity Analysis

Parameter	Intervention	Baseline value	Range for sensitivity analysis	Distribution used in sensitivity analysis	Source
General model					
Start age, y	NA	10	4 to 18	NA	NA
Discount rate, %	NA	3	0 to 6	NA	Hutubessy et al,^42^ 2003
Time horizon, y	NA	5	2 to 10	NA	Hardy et al,^44^ 2013; Brennan et al,^29^ 2021
Total annual costs, Hong Kong $ (US $)	Atropine eye drops, 0.05%	8415 (1072)	5055 to 13 376 (644 to 1704)	Gamma	CUHK Eye Center, Hospital Authority of HKSAR,^40^ 2013
	Atropine eye drops, 0.01%	8439 (1075)	5087 to 13 321 (648 to 1697)	Gamma	CUHK Eye Center, Hospital Authority of HKSAR,^40^ 2013
	DIMS	10 527 (1341)	7214 to 13 149 (919 to 1675)	Gamma	CUHK Eye Center, Hospital Authority of HKSAR,^40^ 2013
	Daily disposable contact lenses	15 064 (1919)	8808 to 17 772 (1122 to 2264)	Gamma	CUHK Eye Center, Hospital Authority of HKSAR,^40^ 2013
	Orthokeratology	19 013 (2422)	15 535 to 21 093 (1979 to 2687)	Gamma	CUHK Eye Center, Hospital Authority of HKSAR,^40^ 2013
	BSLs	8062 (1027)	4318 to 12 748 (550 to 1624)	Gamma	CUHK Eye Center, Hospital Authority of HKSAR,^40^ 2013
	PALs	11 799 (1503)	4851 to 12 913 (618 to 1645)	Gamma	CUHK Eye Center, Hospital Authority of HKSAR,^40^ 2013
	RGPCLs	12 261 (1562)	6288 to 14 695 (801 to 1872)	Gamma	CUHK Eye Center, Hospital Authority of HKSAR,^40^ 2013
	MSCLs	14 099 (1796)	7936 to 16 721 (1011 to 2130)	Gamma	CUHK Eye Center, Hospital Authority of HKSAR,^40^ 2013
	HALs	9907 (1262)	6272 to 14 648 (799 to 1866)	Gamma	CUHK Eye Center, Hospital Authority of HKSAR,^40^ 2013
	Outdoor activity	5888 (750)	0 to 10 158 (0 to 1345)	Gamma	CUHK Eye Center, Hospital Authority of HKSAR,^40^ 2013
	SVLs	5888 (750)	3234 to 10 158 (412 to 1294)	Gamma	CUHK Eye Center, Hospital Authority of HKSAR,^40^ 2013
	Red light therapy	16 147 (2057)	12 921 to 19 374 (1646 to 2468)	Gamma	Eyerising International
Median hourly wage of an adult, HK $ (US $)	NA	152 (19)	86 to 393 (11 to 50)	NA	Census and Statistics Department of HKSAR,^39^ 2021
SER, D	Atropine eye drops, 0.05%	0.57	0.28 to 0.86	Normal	Zhang et al,^30^ 2023
	Atropine eye drops, 0.01%	0.33	0.15 to 0.52	Normal	Zhang et al,^30^ 2023
	DIMS	0.28	−0.24 to 0.80	Normal	Zhang et al,^30^ 2023
	Daily disposable contact lenses	0.25	0.07 to 0.43	Normal	Yu et al,^43^2022
	Orthokeratology	NA	NA	NA	NA
SER, D	BSLs	−0.07	−0.38 to 0.23	Normal	Zhang et al,^30^ 2023
	PALs	0.13	−0.07 to 0.33	Normal	Zhang et al,^30^ 2023
	RGPCLs	0.30	−0.01 to 0.62	Normal	Zhang et al,^30^ 2023
	MSCLs	0.26	−0.25 to 0.77	Normal	Zhang et al,^30^2023
	HALs	0.34	−0.79 to 1.50	Normal	Zhang et al,^30^ 2023
	Outdoor activity	0.16	−0.01 to 0.35	Normal	Zhang et al,^30^ 2023
	SVLs	−0.81	−0.71 to −0.91	Normal	Yam et al,^11^ 2019
	Red light therapy	0.59	0.06 to 1.10	Normal	Zhang et al,^30^ 2023
AL, mm	Atropine eye drops, 0.05%	−0.30	−0.65 to 0.05	Normal	Zhang et al,^30^ 2023
	Atropine eye drops, 0.01%	−0.17	−0.38 to 0.04	Normal	Zhang et al,^30^ 2023
	DIMS	−0.16	−0.78 to 0.46	Normal	Zhang et al,^30^ 2023
	Daily disposable contact lenses	−0.12	−0.25 to 0.01	Normal	Yu et al,^43^ 2022
	Orthokeratology	−0.36	−0.53 to −0.20	Normal	Zhang et al,^30^ 2023
	BSLs	−0.07	−0.50 to 0.38	Normal	Zhang et al,^30^ 2023
	PALs	0.15	−0.13 to 0.44	Normal	Zhang et al,^30^ 2023
	RGPCLs	−0.05	−0.42 to 0.31	Normal	Zhang et al,^30^ 2023
	MSCLs	−0.07	−0.69 to 0.56	Normal	Zhang et al,^30^ 2023
	HALs	−0.17	−0.94 to 0.61	Normal	Zhang et al,^30^ 2023
	Outdoor activity	−0.10	−0.35 to 0.15	Normal	Zhang et al,^30^ 2023
	SVLs	0.41	0.09 to 0.46	Normal	Yam et al,^11^ 2019
	Red light therapy	−0.25	−0.86 to 0.39	Normal	Zhang et al,^30^ 2023
Probabilities of adverse events					
Photophobia	NA	0.08	NA	NA	Yam et al,^11^ 2019
Allergic conjunctivitis	NA	0.03	NA	NA	Yam et al,^11^ 2019
Microbial keratitis	NA	0.0003	NA	NA	Bullimore et al,^45^ 2019
Corneal infiltrate	NA	0.004	NA	NA	Bullimore et al,^45^ 2019
Ocular allergies (contact lenses)	NA	0.14	NA	NA	Gaume Giannoni et al,^38^ 2022
Hordeolum	NA	0.07	NA	NA	Gaume Giannoni et al,^38^ 2022
Corneal staining	NA	0.28	NA	NA	Gaume Giannoni et al,^38^ 2022

Abbreviations: AL, axial length; BSLs, bifocal spectacle lenses; CUHK, Chinese University of Hong Kong; D, diopter; DIMS, defocus incorporated multiple segment spectacles; HALs, highly aspherical lenslets; HKSAR, Hong Kong Special Administrative Region; MSCLs, multifocal soft contact lenses; NA, not applicable; PALs, progressive addition lenses; RGPCLs, rigid gas-permeable contact lenses; SER, spherical equivalent refraction; SVLs, single-vision lenses.

## Results

**Figure 1.**
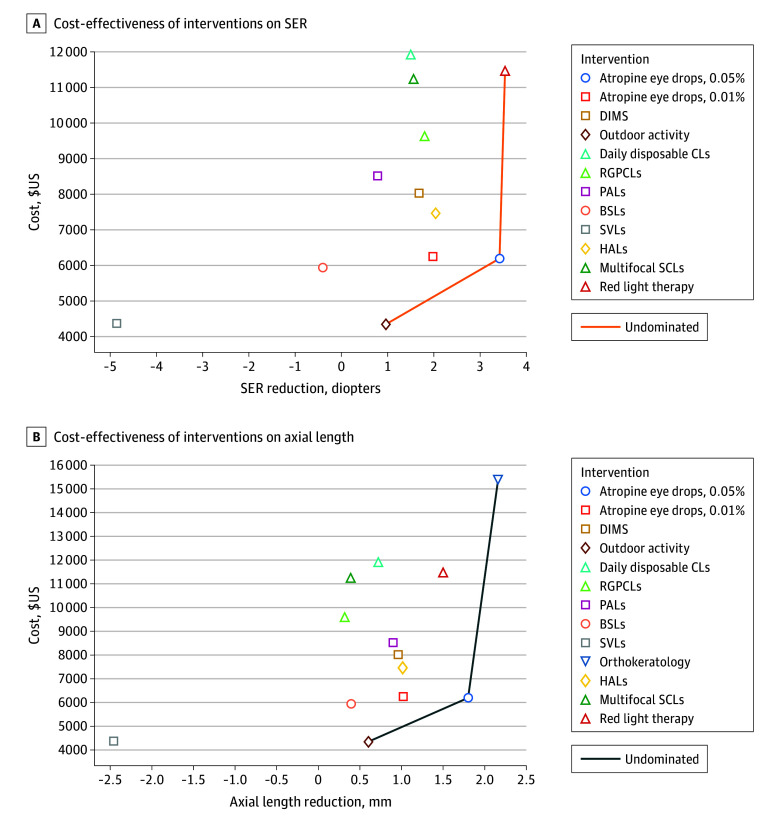
Cost-Effectiveness Analysis Plane BSLs indicates bifocal spectacle lenses; DIMS, defocus incorporated multiple segment spectacles; HALs, highly aspherical lenslets; SCLs, soft contact lenses; PALs, progressive addition lenses; RGPCLs, rigid gas-permeable contact lenses; SER, spherical equivalent refraction; SVLs, single-vision lenses.

Figure 1 and Table 2 show the results of the base-case analysis. The projected total cost of treating myopia progression over a 5-year time horizon was least for outdoor activities, with a total cost of HK $34 108 (US $4345). Over this period, orthokeratology was the most expensive, with a total cost of HK $120 474 (US $15 347), followed by daily disposable contact lenses (HK $93 588 [US $11 922]), red light therapy (HK $90 102 [US $ 11 478]), and MSCLs (HK $88 313 [US $11 250]). Compared with a patient who is not treated (ie, given only SVL), a patient who was treated with outdoor activity resulted in an incremental cost saving of HK $204 (US $26). Similarly, low-dose atropine eye drops (ie, 0.05% and 0.01%) accrued fewer additional costs than contact lens options. Their incremental costs were HK $14 303 (US $1822) for the 0.05% dose and HK $14 750 (US $1879) for the 0.01% dose.

**Table 2.** Base-Case Cost-Effectiveness Results

Intervention	Cost, Hong Kong $ (US $)	Incremental cost, Hong Kong $ (US $)	Effectiveness^a^	Incremental effectiveness^a^	ICER, Hong Kong $ (US $)/reduction
**Spherical equivalent refraction**					
SVLs	34 320 (4372)	NA	−4.86	NA	NA
BSLs	46 621 (5939)	12 301 (1567)	−0.40	4.46	2763 (352)
DIMS	63 036 (8030)	28 715 (3658)	1.68	6.54	4388 (559)
Atropine eye drops, 0.01%	49 070 (6251)	14 750 (1879)	1.98	6.84	2159 (275)
Daily disposable CLs	93 588 (11 922)	56 268 (7550)	1.50	6.36	9318 (1187)
RGPCLs	75 517 (9620)	41 197 (5248)	1.80	6.66	6186 (788)
HALs	58 592 (7464)	24 272 (3092)	2.04	6.90	3517 (448)
MSCLs	88 313 (11 250)	54 000 (6879)	1.56	6.42	8407 (1071)
PALs	66 858 (8517)	32 538 (4145)	0.78	5.64	5770 (735)
Outdoor	34 108 (4345)	−204 (−26)	0.96	5.82	−39 (−5)^b^
Atropine eye drops, 0.05%	43 615 (6193)	14 303 (1822)	3.42	8.28	1727 (220)^b^
Red light therapy	90 102 (11 478)	55 782 (7106)	3.54	8.40	6641 (846)^b^
**Axial length**					
SVLs	34 320 (4372)	NA	−2.46	NA	NA
BSLs	46 621 (5939)	12 3-01 (1567)	0.40	2.86	4302 (548)
DIMS	63 036 (8030)	28 715 (3658)	0.96	3.42	8400 (1070)
Atropine, eye drops, 0.01%	49 070 (6251)	14 750 (1879)	1.02	3.48	4239 (540)
Daily disposable CLs	93 588 (11 922)	59 268 (7550)	0.72	3.18	18 636 (2374)
RGPCLs	75 517 (9620)	41 197 (5248)	0.32	2.78	14 821 (1888)
HALs	58 592 (7464)	24 272 (3092)	1.02	3.48	6979 (889)
MSCLs	88 313 (11 250)	54 000 (6879)	0.39	2.85	18 950 (2414)
PALs	66 858 (8517)	32 538 (4145)	0.90	3.36	9687 (1234)
Red light therapy	90 102 (11 478)	55 782 (7106)	1.50	3.96	14 0831794
Orthokeratology	120 474 (15 347)	86 154 (10 975)	2.16	4.62	18 652 (2376)^b^
Outdoor	34 108 (4345)	−204 (−26)	0.60	3.06	−63 (−8)^b^
Atropine eye drops, 0.05%	43 615 (6193)	14 303 (1822)	1.80	4.26	3360 (428)^b^

Abbreviations: BSLs, bifocal spectacle lenses; CLs, contact lenses; DIMS, defocus incorporated multiple segment spectacles; HALs, highly aspherical lenslets; ICER, incremental cost-effectiveness ratio; MSCLs, multifocal soft CLs; NA, not applicable; PALs, progressive addition lenses; RGPCLs, rigid gas-permeable CLs; SVLs, single-vision lenses.

^a^ Unit of measure is diopters for spherical equivalent refraction and millimeters for axial length.

^b^ Dominant strategies.

Regarding the SER (Figure 1A), outdoor activity, atropine, 0.05%, and red light therapy were cost-effective. The ICER of atropine, 0.05%, was a HK $1727 (US $220)/SER reduction and the ICER of red light therapy was HK $6641 (US $846)/SER reduction, with outdoor activity yielding a cost-savings of HK $39 (US $5)/SER reduction. For AL (Figure 1B), outdoor activity, atropine, 0.05%, and orthokeratology were cost-effective. The ICER of atropine, 0.05%, was HK $3360 (US $428)/AL reduction and the ICER of orthokeratology was HK $18 652 (US $2376)/AL reduction, with outdoor activity yielding a cost savings of HK $63 (US $8)/AL reduction. The ICERs of the spectacle options (DIMS, BSLs, PALs, and HALs) ranged from HK $2763 (US $352)/SER reduction to HK $5770 (US $735)/SER reduction and HK $4302 (US $548)/AL reduction to HK $9687 (US $1234)/AL reduction. The ICERs of contact lenses (daily disposable contact lenses, MSCLs, RGPCLs, and orthokeratology) ranged from HK $6186 (US $788)/SER reduction to HK $9318 (US $1187)/SER reduction and HK $14 821 (US $1888)/AL reduction to HK $18 950 (US $2414)/AL reduction. The ICERs of contact lenses (daily disposable contact lenses, MSCLs, RGPCLs, and orthokeratology) ranged from HK $6186 (US $788)/SER reduction to HK $9318 (US $1187)/SER reduction and HK $14 821 (US $1888)/AL reduction to HK $18 950 (US $2414)/AL reduction. We conducted several sensitivity analyses within plausible ranges of major model parameters to determine their effect on the model. The results show that changes in the costs of the interventions and SER had the largest influence on the cost-effectiveness results (eFigure 2 in Supplement 1).

**Figure 2.**
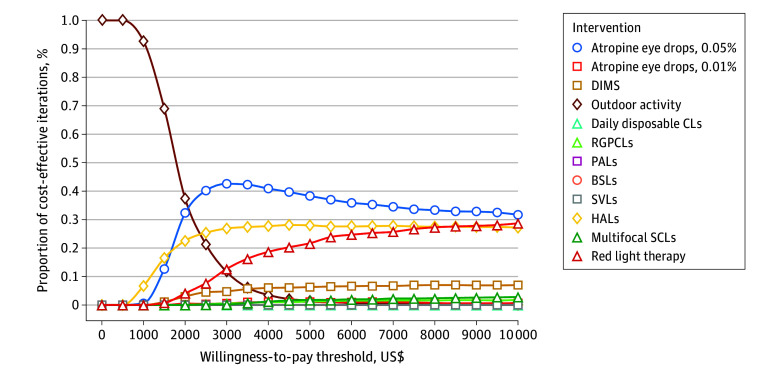
Probabilistic Sensitivity Analysis Cost-effectiveness acceptability curves are shown for interventions in terms of spherical equivalent refraction. The dotted lines represent the probability of each strategy being cost-effective at willingness-to-pay thresholds of US $0/SER reduction to US $10 000/SER reduction. BSLs indicates bifocal spectacle lenses; DIMS, defocus incorporated multiple segment spectacles; HALs, highly aspherical lenslets; SCLs, soft contact lenses; PALs, progressive addition lenses; RGPCLs, rigid gas-permeable contact lenses; SVLs, single-vision lenses.

Additionally, we performed probabilistic sensitivity analysis to determine the optimum strategy at varying WTPs in a Monte Carlo simulation, using 1000 iterations (Figure 2 and eFigure 3 in Supplement 1). Atropine, 0.05%, was the optimal strategy at WTP thresholds of HK $19 625 (US $2500) (SER) to HK $27 475 (US $3500) (AL) and beyond. However, outdoor activity became the optimal strategy below these thresholds. At a WTP of HK $0 (US $0)/SER reduction to HK $3925 ($500)/SER reduction, outdoor activity was 100% cost-effective. The iterations for outdoor activity to be cost-effective reduced as the WTP increased. This decreased to 92% when WTP was HK $7850 (US $1000)/SER reduction, 69% when WTP was HK $11 775 (US $1500)/SER reduction, and 37% when WTP was HK $15 700 (US $2000)/SER reduction. Beyond this threshold, outdoor activity was less likely to be cost-effective. At a WTP of HK $19 625 (US $2500)/SER reduction, atropine, 0.05%, was about 40% cost-effective. At this threshold, HALs and red light therapy were about 25% and 8% cost-effective, respectively. At the highest estimated WTP (ie, HK $78 500 [US $10 000]/SER reduction), atropine, 0.05%, remained the most likely cost-effective intervention with iterations of 32%, followed by red light therapy and HALs, with iterations of 29% and 27%, respectively. A similar trend was observed regarding AL (eFigure 3 in Supplement 1). At lower WTP thresholds, outdoor activity was the most likely cost-effective strategy, while atropine, 0.05%, became the most likely cost-effective strategy as the WTP increased. At a WTP of HK $27 475 (US $3500)/AL reduction, BSLs and DIMS were about 12% and 11% cost-effective, respectively. At the highest WTP threshold (HK $78 500 [US $ 10 000/AL reduction]), DIMS were about 15% cost-effective.

## Discussion

To our knowledge, this economic evaluation is one of the first studies to demonstrate the cost-effectiveness of interventions for myopia progression. We included 13 interventions, providing a comprehensive assessment of a wide range of interventions for myopia progression over a 5-year time horizon. Our results show that at higher WTP thresholds (HK $19 625 [US $2500]/SER reduction to HK $78 500 [US $10 000]/SER reduction), atropine, 0.05%, was the strategy most likely to be cost-effective, followed by HALs and red light therapy. Below this threshold, outdoor activity was the most likely cost-effective strategy. Our study provides information for policy makers in addressing myopia.

The iterations of atropine, 0.05%, to be cost-effective was about 40% at a WTP of HK $19 625 (US $2500)/SER reduction, compared with 25% for HALs and 8% for red light therapy. The iterations of red light therapy to be cost-effective increased as the WTP threshold increased. Although red light therapy could be cost-effective (29%) at higher WTP thresholds (HK $78 500 [US $10 000]/SER reduction), it is more costly than atropine, 0.05%.

In terms of AL, atropine, 0.05%, was the most likely cost-effective strategy at a WTP threshold of HK $27 475 (US $3500) and beyond. Below this threshold, outdoor activity remained the most likely cost-effective intervention. Orthokeratology was a dominant strategy in the base-case analysis, meaning it was more effective than the reference case strategy. However, atropine, 0.05%, was more likely to be cost-effective than orthokeratology in the probabilistic analysis, possibly due to the high costs associated with orthokeratology.

Outdoor activity was not associated with any significant cost and was the least expensive strategy, while contact lenses (orthokeratology, daily disposable contact lenses, MSCLs, and RGPCLs) were the most expensive strategies. In addition to the benefit of reducing myopia onset and progression in children,^46,47,48,49,50,51^ outdoor activity also improves their general health and development,^52^ reduces childhood obesity,^53^ prevents chronic disease, and increases vitamin D levels for development of bones and teeth.^54,55^ Given these health benefits, measures to encourage and promote outdoor activities should be strongly advocated. Orthokeratology is associated with other difficulties such as the need for skills for contact lens fitting, the risk of infective keratitis, and pain. These factors have limited the widespread use of orthokeratology.^25^ Although orthokeratology could be cost-effective, it requires strict monitoring due to the potential sight-threatening complications.

Notably, although atropine, 0.05%, and outdoor activity are the most likely cost-effective interventions, the other interventions included in this study could be cost-effective if they were modeled independently with SVLs. Atropine, 0.05%, and outdoor activity are less expensive, hence emerging as the most likely cost-effective interventions. Additionally, these other interventions were associated with a certain level of cost-effectiveness at varying WTP thresholds. For instance, at a WTP of HK $27 475 (US $3500)/AL reduction, BSLs and DIMS were about 12% and 11% cost-effective, respectively. At the highest WTP threshold (HK $78 500 [US $ 10 000]/AL reduction), DIMS were about 15% cost-effective.

Additionally, the World Health Organization defines an intervention as highly cost-effective if it costs less than the per-capita gross domestic product (GDP) and as cost-effective if it costs less than 3 times the per capita GDP for a given country.^42^ In this study, the ICERs of the spectacle options (DIMS, BSLs, PALs, and HALs) ranged from HK $2763 (US $352)/SER reduction to HK $5770 (US $735)/SER reduction and HK $4302 (US $548)/AL reduction to HK $9687 (US $1234)/AL reduction, which were below the per-capita GDP of Hong Kong (HK $382 377 [US $48 710]/annum in 2022).^56^ Similarly, the ICERs of contact lenses (daily disposable contact lenses, MSCLs, RGPCLs, and orthokeratology) ranged from HK $6186 (US $788)/SER reduction to HK $9318 (US $1187)/SER reduction and HK $14 821 (US $1888)/AL reduction to HK $18 950 (US $2414)/AL reduction, which were below the per-capita GDP of Hong Kong. Nevertheless, the comparison is important, as there are several options for the treatment of myopia progression. Moreover, it is important to know which intervention offers the best economic value.

### Limitations

Our study has some limitations. First, costs associated with interventions may have been underestimated. Even though we adapted a societal perspective, certain indirect costs (eg, cost of transportation) were not included in our analysis. Second, costs were calculated according to the charges of the Chinese University of Hong Kong Eye Centre. The results of our study may not be generalizable to other regions that do not have a similar economic setting as Hong Kong. It is worth noting that the costs involved may vary considerably in different regions. Third, we did not assess the impact of myopia interventions on quality of life. The use of orthokeratology compared with SVLs and soft contact lenses, as well as low-concentration atropine compared with SVLs, showed comparable effects on quality of life.^45^ However, a reduction in myopia progression by 1 D could significantly affect future occurrence of pathologic myopia.^44^ Because myopia interventions are intended to prevent progression to pathological myopia, effectiveness was assessed based on their capacity to reduce SER and AL. Fourth, the potential for rebound with myopia interventions has not been accounted for. Fifth, efficacy data were obtained from published meta-analyses, therefore treatment efficacy may vary between different regions; however, our sensitivity analyses accounted for these variations. Last, our model did not account for a pathological state of myopia. Pathologic myopia is a distinct condition that differs from regular myopia. The primary objective of interventions aimed at myopia progression is generally to reduce the progression of myopia, rather than treating the complications that are associated with pathologic myopia. Effective myopia progression interventions may not necessarily treat pathologic myopia complications. Moreover, limited data are available on the effect of myopia interventions on pathologic myopia because of the shorter duration of randomized clinical trials. Additionally, we modeled our study over a time horizon of 5 years, by which time a 10-year-old child (start age) would not have developed pathologic myopia.

## Conclusions

The findings of this economic evaluation suggest that atropine, 0.05%, and outdoor activity may be cost-effective approaches for controlling myopia progression in children. Though more expensive, red light therapy, HALs, and orthokeratology may also be cost-effective. The use of these interventions could help control myopia in a cost-effective way.

## Notes

Supplement 1. **eMethods.** Transition Probabilities (TPs) **eTable.** Sources and Characteristics of Studies for Transition Probabilities **eFigure 1.** Markov Model **eFigure 2.** Deterministic Sensitivity Analysis (Tornado Plots) **eFigure 3.** Cost-Effectiveness Acceptability Curves in Terms of Axial Length
eReferences

Supplement 2.
Data Sharing Statement
