# Error in Discussion

**DOI:** 10.1001/jamanetworkopen.2025.18553

**Published:** 2025-05-28

In the Original Investigation titled “Cost-Effectiveness Analysis of Myopia Progression Interventions in Children,”^1^ published November 2, 2023, the per-capita gross domestic product value for Hong Kong in 2022 and the US equivalent were incorrect. This article has been corrected.
