# Supplementary material for: Cost-Effectiveness Analysis of Myopia Progression Interventions in Children
Source: JAMA Netw Open. 2023 Nov 2;6(11):e2340986. doi: 10.1001/jamanetworkopen.2023.40986 (PMC10623196; doi:10.1001/jamanetworkopen.2023.40986)
Supplement: Supplement 1. — eMethods. Transition Probabilities (TPs) eTable. Sources and Characteristics of Studies for Transition Probabilities eFigure 1. Markov Model eFigure 2. Deterministic Sensitivity Analysis (Tornado Plots) eFigure 3. Cost-Effectiveness Acceptability Curves in Terms of Axial Length eReferences [file jamanetwopen-e2340986-s001.pdf]

Supplementary Online Content

Agyekum S, Chan PP, Adjei PE, et al. Cost-effectiveness analysis of myopia progression interventions in children. *JAMA Netw Open*. 2023;6(11):e2340986. doi:10.1001/jamanetworkopen.2023.40986

eMethods. Transition Probabilities (TPs)
eTable. Sources and Characteristics of Studies for Transition Probabilities
eFigure 1. Markov Model
eFigure 2. Deterministic Sensitivity Analysis **(**Tornado Plots)
eFigure 3. Cost-Effectiveness Acceptability Curves in Terms of Axial Length
eReferences

This supplementary material has been provided by the authors to give readers additional information about their work.

© 2023 Agyekum S et al. *JAMA Network Open*.

eMethods. Transition Probabilities (TPs)

To calculate the realistic transition probabilities between health states in our model,[1] we used the rates of myopia progression from randomized controlled trials and were categorized as slow, intermediate, or rapid progression, defined as <0.5 D, 0.5 to 0.99 D, and ≥1.0 D, respectively.[2–4]

Given two health states i and j, let $\gamma_{i,j}$ represent the optimum rate of progression from i to j. Similarly, let $\pi_{i,j}(t)$ represent the calculated TP from i to j over a period t. Let G represent the matrix of optimum rates of progression between health states; with elements $\gamma_{i,j}$, and $P(t)$ represent a matrix of corresponding transition probabilities with elements $\pi_{i,j}(t)$. The relationship between $P(t)$ and G is shown in equation 1.[5]

$$P(t) = \exp(tG) = \sum_{n=0}^{\infty} \frac{t^n}{n!} G^n \qquad \text{Equation 1}$$

The sum of transition rates from state λ_i is given in equation 2, the solution to equation 1 in our context, i.e., a three-state model with forward transitions only, is given in equation 3

$$\lambda_i = \sum_{j \neq i} \gamma_{i,j} \qquad \text{Equation 2}$$

$$P(t) = \begin{pmatrix} \pi_{1,1}(t) & \pi_{1,2}(t) & \pi_{1,3}(t) \\ \pi_{2,1}(t) & \pi_{2,2}(t) & \pi_{2,3}(t) \\ \pi_{3,1}(t) & \pi_{3,2}(t) & \pi_{3,3}(t) \end{pmatrix} = \begin{pmatrix} e^{-\lambda_1 t} & \frac{\gamma_{1,2}(e^{-\lambda_2 t} - e^{-\lambda_1 t})}{\lambda_1 - \lambda_2} & (1 - \pi_{1,1}(t) - \pi_{1,2}(t)) \\ 0 & e^{-\lambda_2 t} & (1 - \pi_{2,1}(t) - \pi_{2,2}(t)) \\ 0 & 0 & 1 \end{pmatrix} \qquad \text{Equation 3}$$

© 2023 Agyekum S et al. *JAMA Network Open*.

eTable. Sources and Characteristics of Studies for Transition Probabilities

Author	Study area	Sample size	Follow-up (years)	Age (years)	Intervention	Baseline SE (D)	Baseline AL (mm)	Change in SE (D/year)	Change in AL (mm/year)
Yam et al, 2018[2]	Hong Kong	438	1	4-12	Atropine 0.05%	-3.98 (1.69)	24.85 (0.90)	-0.27 (0.61)	0.20 (0.25)
					Placebo	-3.85 (1.95)	24.82 (0.97)	-0.81 (0.53)	0.41 (0.22)
Yam et al, 2018[2]	Hong Kong	438	1	4-12	Atropine 0.01%	-3.77 (1.85)	24.70 (0.99)	-0.59 (0.61)	0.36 (0.29)
					Placebo	-3.85 (1.95)	24.82 (0.97)	-0.81 (0.53)	0.41 (0.22)
Fulk et al, 2000[6]	United States	82	2.5	6-12.9	Bifocal	-2.12 (1.16)	24.02 (0.78)	0.40 (0.35)	NA
					SVL	-2.52 (1.40)	24.10 (0.71)	0.50 (0.33)	NA
Lam et al, 2020[7]	Hong Kong	183	2	8-13	DIMS	-2.97 (0.97)	24.70 (0.82)	-0.17 (0.05)	0.11 (0.02)
					SVL	-2.76 (0.96)	24.60 (0.83)	-0.55 (0.04)	0.32 (0.02)
Bao et al, 2022[8]	China	170	2	8-13	HAL	-2.70 (0.14)	24.76 (0.09)	-0.27 (0.06)	0.13 (0.02)
					SAL	-2.31 (0.13)	24.43 (0.10)	-0.48 (0.05)	0.25 (0.02)
					SVL	-2.46 (0.12)	24.77 (0.09)	-0.81 (0.06)	0.36 (0.02)
Gwiazda and Chandler, 2011[9]	United States	118	3	8- <12	PAL	-1.50 (0.45)	NA	-0.29 (0.39)	NA
					SVL	-1.45 (0.47)	NA	-0.42 (0.37)	NA
Katz et al, 2003[10]	Singapore	428	2	6-12	RGP	-2.84 (0.83)	24.39 (0.79)	-0.65 (0.55)	0.35 (0.41)
					SVL	-2.63 (0.85)	24.46 (0.79)	-0.63 (0.49)	0.33 (0.40)
Cho & Cheung, 2012[4]	Hong Kong	102	2	6-10	Orthokeratology	-2.05 (0.72)	24.48 (0.71)	NA	0.20 (0.15)
					SVL	-2.23 (0.84)	24.40 (0.84)	NA	0.37 (0.16)
					SVL	-1.75 0.94	24.00 (0.86)	-0.44 (0.28)	0.24 (0.11)
Chamberlain et al, 2019[11]	Multicenter	144	3	8-12	MiSight Lenses	-2.02 (0.77)	24.42 (0.66)	-0.18 (0.39)	0.09 (0.13)
					Proclear (control)	-2.19 (0.81)	24.46 (0.70)	-0.58 (0.41)	0.24 (0.15)
Sankaridurg et al, 2019[12]	China	508	3	7-13	SHCL I (+2.50)	-2.38 (0.82)	24.70 (0.80)	-0.50 (0.35)	0.21 (0.17)
					SHCL II (+1.50)	-2.39 (0.79)	24.50 (0.70)	-0.53 (0.43)	0.24 (0.17)
					CL III (EDOF – +1.70D)	-2.41 (0.82)	24.50 (0.70)	-0.47 (0.39)	0.22 (0.16)
					CL IV (EDOF – +2.50D)	-2.44 (0.73)	24.60 (0.80)	-0.50 (0.34)	0.22 (0.14)
					Single vision SHCL (control)	-2.29 (0.75)	24.70 (0.80)	-0.66 (0.33)	0.33 (0.14)
					SVL	-1.93 (0.74)	24.28 (0.83)	-1.30 (0.44)	0.72 (0.21)
Wu et al, 2018[3]	Taiwan	693	1	6-7	Outdoor activity	-0.36 (1.14)	22.78 (0.77)	-0.57 (0.40)	0.45 (0.28)
					Control	-0.30 (0.99)	22.81 (0.76)	-0.79 (0.38)	0.60 (0.19)

© 2023 Agyekum S et al. *JAMA Network Open*.

Jiang et al,2022[13]	China	264	1	8-13	Repeated low-level red-light (RLRL)	-2.49 (0.92)	24.54 (0.67)	-0.20 (95% CI, -0.29 to -0.11)	0.13 (95% CI, 0.09-0.17)
					SVL	-2.67 (1.06)	24.62 (0.86)	-0.79 (95% CI, -0.88 to -0.69)	0.38 (95% CI, 0.34-0.42)

SVL; single vision lenses, SCL; soft contact lens, RGP; rigid gas permeable, PAL; progressive addition lens, DIMS; defocus incorporated multiple segment spectacle, HAL; SAL; SHCL; EDOF; enhanced depth of focus; PALs, progressive addition spectacle lenses; RGPs, rigid gas-permeable contact lenses

© 2023 Agyekum S et al. *JAMA Network Open*.

eFigure 1. Markov Model

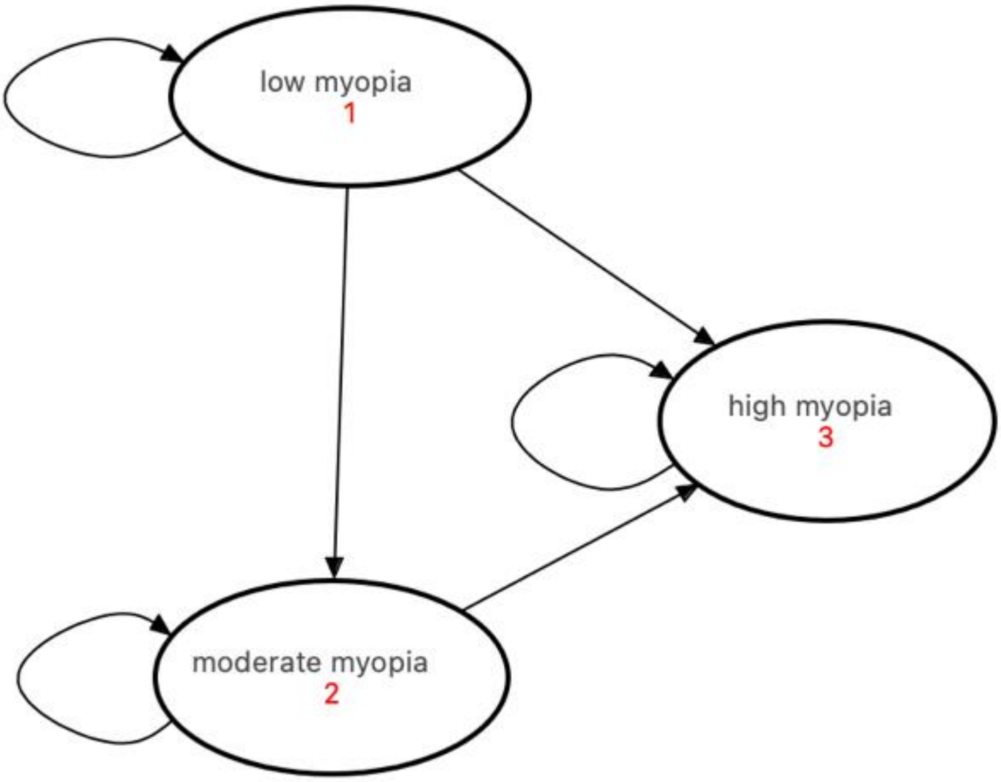

Markov model showing myopia states (ovals) and possible transitions (arrows). Numbers 1, 2, & 3 represent low, moderate, and high myopia respectively. Looped arrows represent remaining in the same myopic state.

© 2023 Agyekum S et al. *JAMA Network Open*.

eFigure 2. Deterministic Sensitivity Analysis (Tornado Plots)

a.

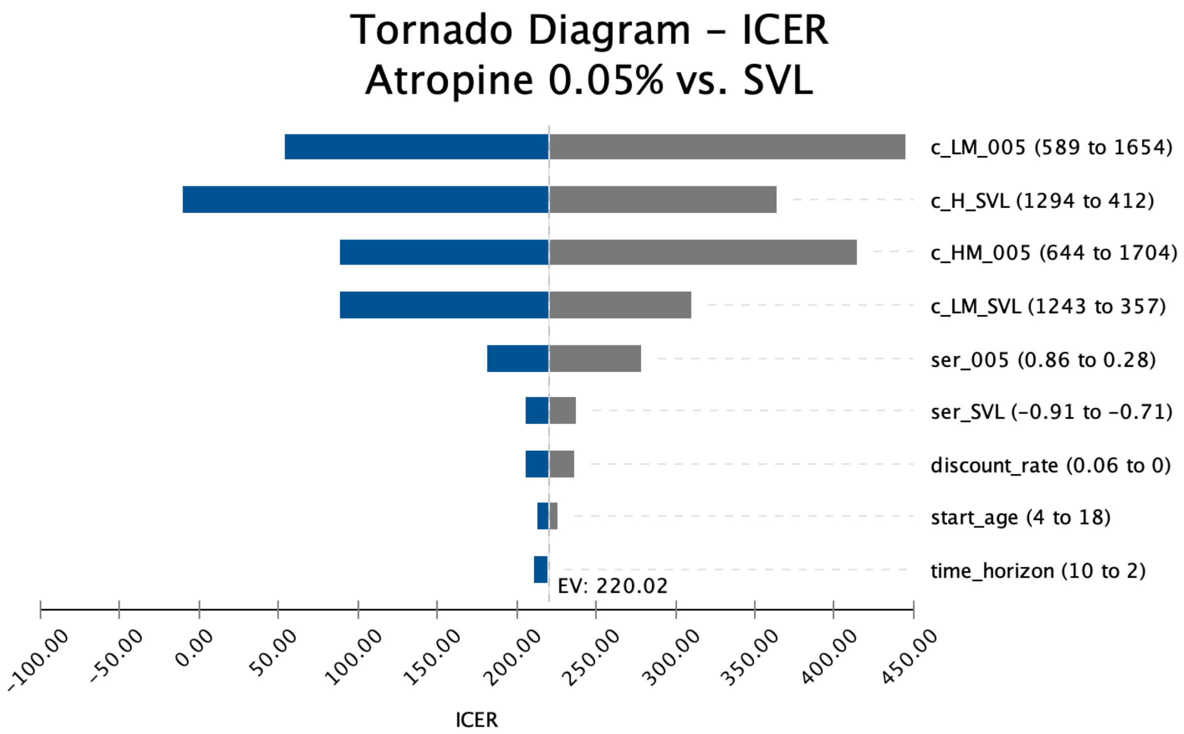

c_LM_005 = cost of atropine 0.05% for low to moderate myopia
c_HM_005 = cost of atropine 0.05% for high myopia
c_ LM_SVL = cost of single vision lenses for low to moderate myopia
c_H_SVL = cost of single vision lenses for high myopia
ser_005 = spherical equivalent refraction of atropine 0.05%
ser_SVL = spherical equivalent refraction of single vision lenses

© 2023 Agyekum S et al. *JAMA Network Open*.

b.

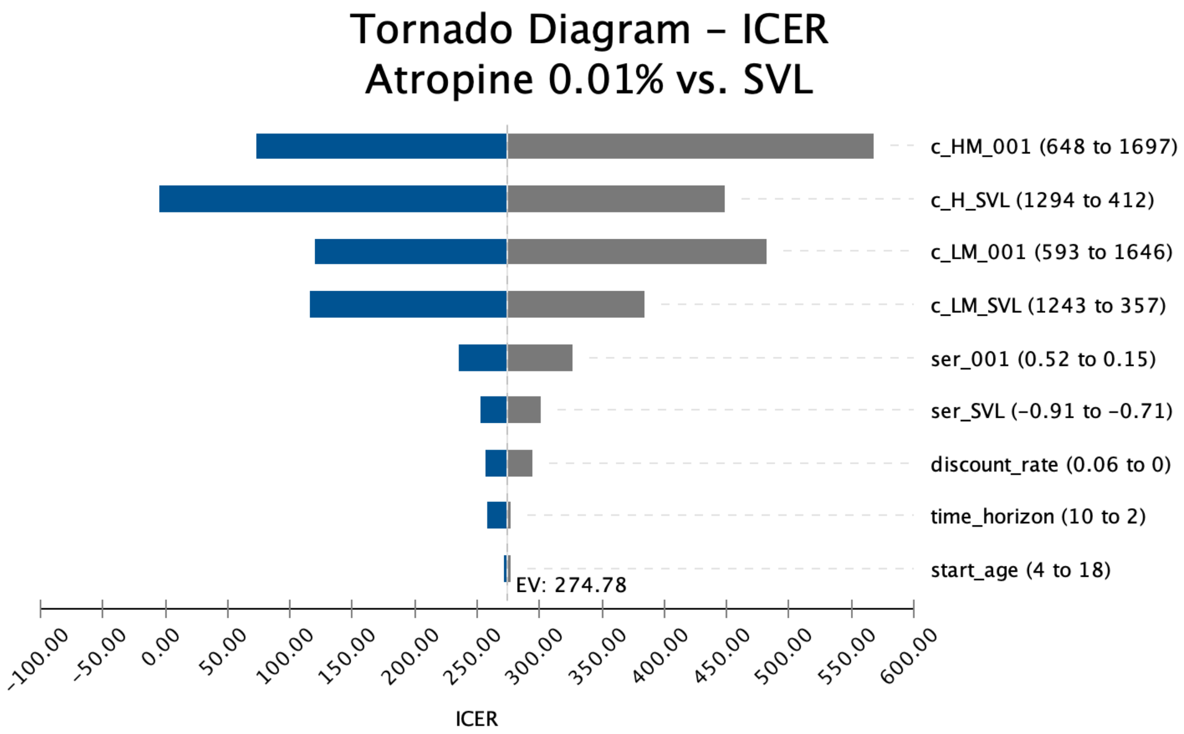

c_LM_001 = cost of atropine 0.01% for low to moderate myopia
c_HM_001 = cost of atropine 0.01% for high myopia
c_ LM_SVL = cost of single vision lenses for low to moderate myopia
c_H_SVL = cost of single vision lenses for high myopia
ser_001 = spherical equivalent refraction of atropine 0.01%
ser_SVL = spherical equivalent refraction of single vision lenses

© 2023 Agyekum S et al. *JAMA Network Open*.

c.

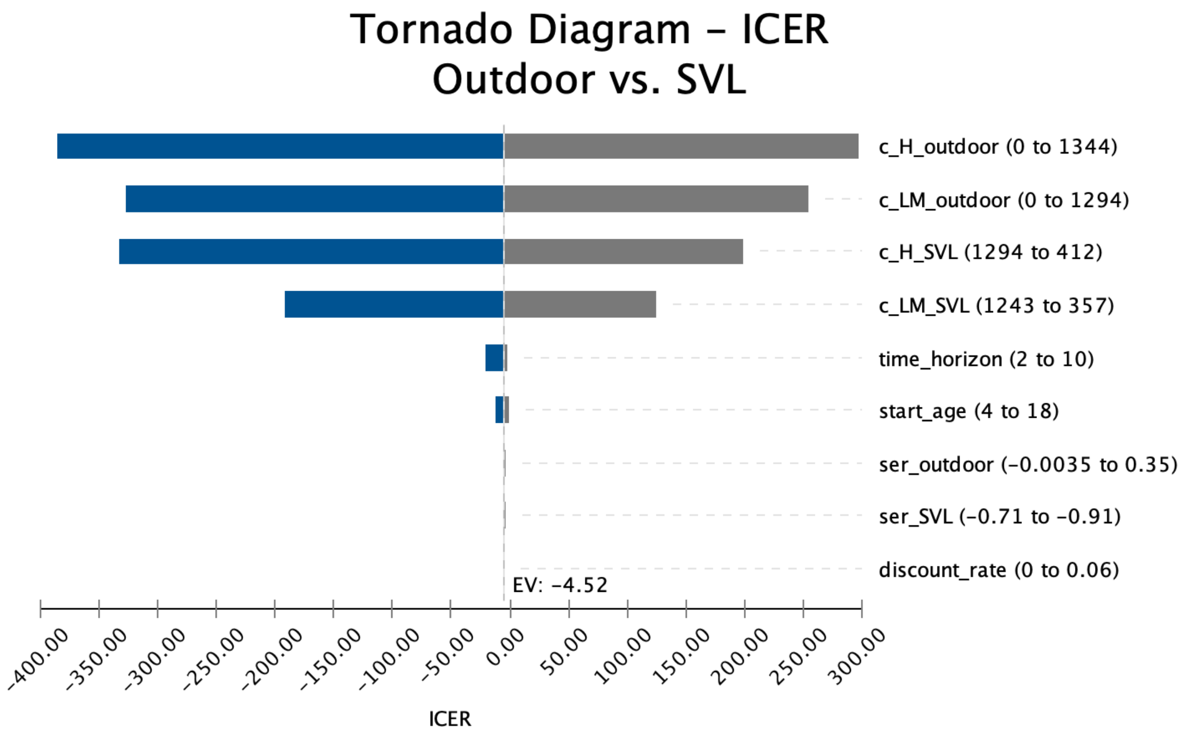

c_LM_outdoor = cost of outdoor activity for low to moderate myopia
c_H_outdoor= cost of outdoor activity for high myopia
c_ LM_SVL = cost of single vision lenses for low to moderate myopia
c_H_SVL = cost of single vision lenses for high myopia
ser_outdoor = spherical equivalent refraction of outdoor activity
ser_SVL = spherical equivalent refraction of single vision lenses

© 2023 Agyekum S et al. *JAMA Network Open*.

d.

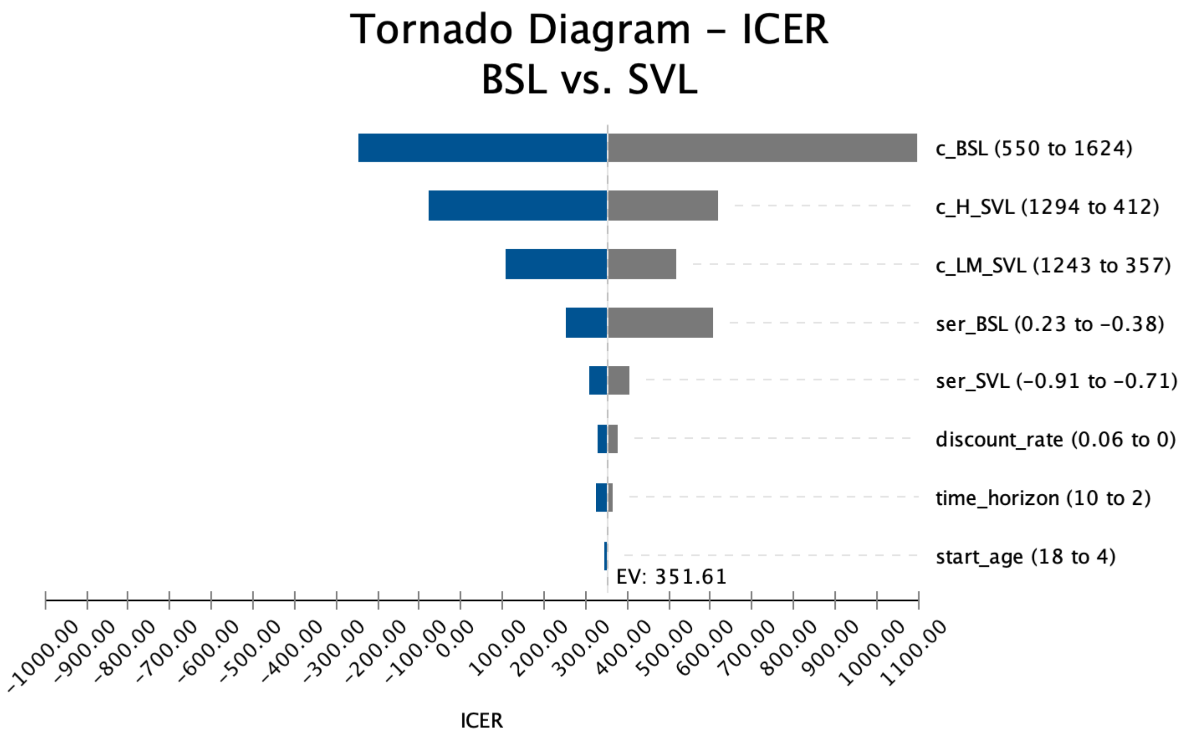

c_BSL = cost of bifocal spectacle lenses (BSL)
c_ LM_SVL = cost of single vision lenses for low to moderate myopia
c_H_SVL = cost of single vision lenses for high myopia
ser_BSL = spherical equivalent refraction of BSL
ser_SVL = spherical equivalent refraction of single vision lenses

© 2023 Agyekum S et al. *JAMA Network Open*.

e.

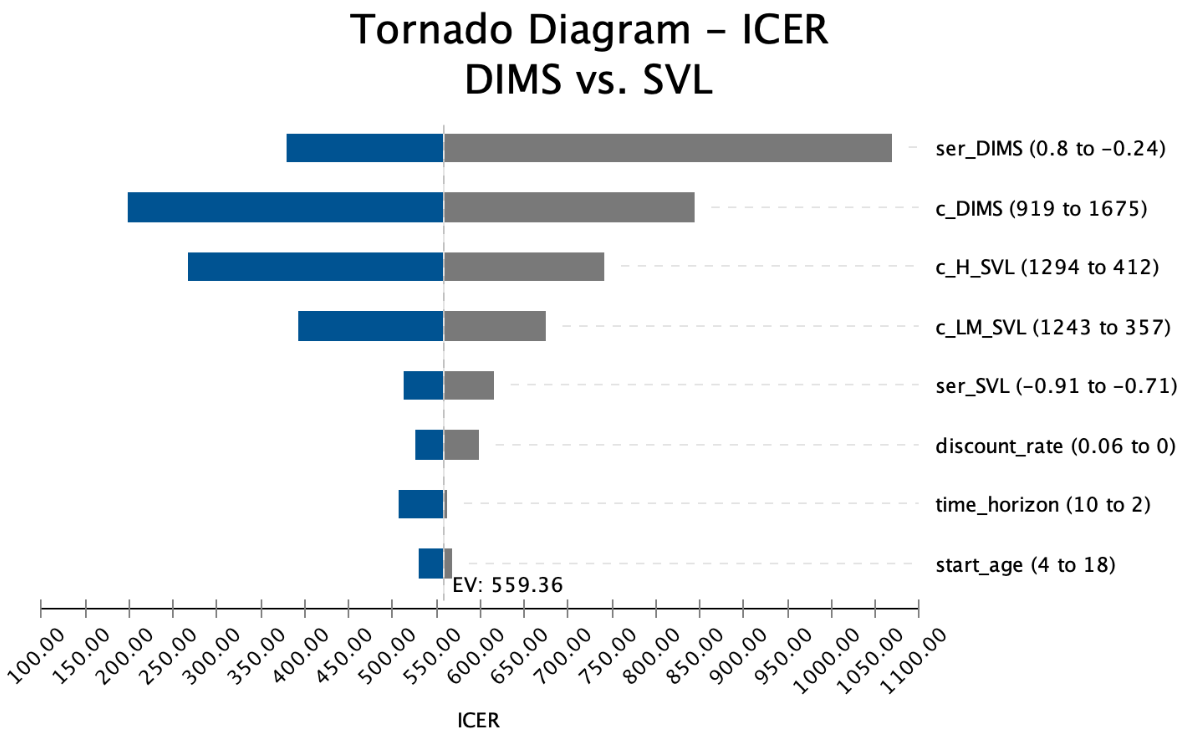

c_DIMS = cost of defocus incorporated multiple segment spectacles (DIMS)
c_ LM_SVL = cost of single vision lenses for low to moderate myopia
c_H_SVL = cost of single vision lenses for high myopia
ser_DIMS = spherical equivalent refraction of DIMS
ser_SVL = spherical equivalent refraction of single vision lenses

© 2023 Agyekum S et al. *JAMA Network Open*.

f.

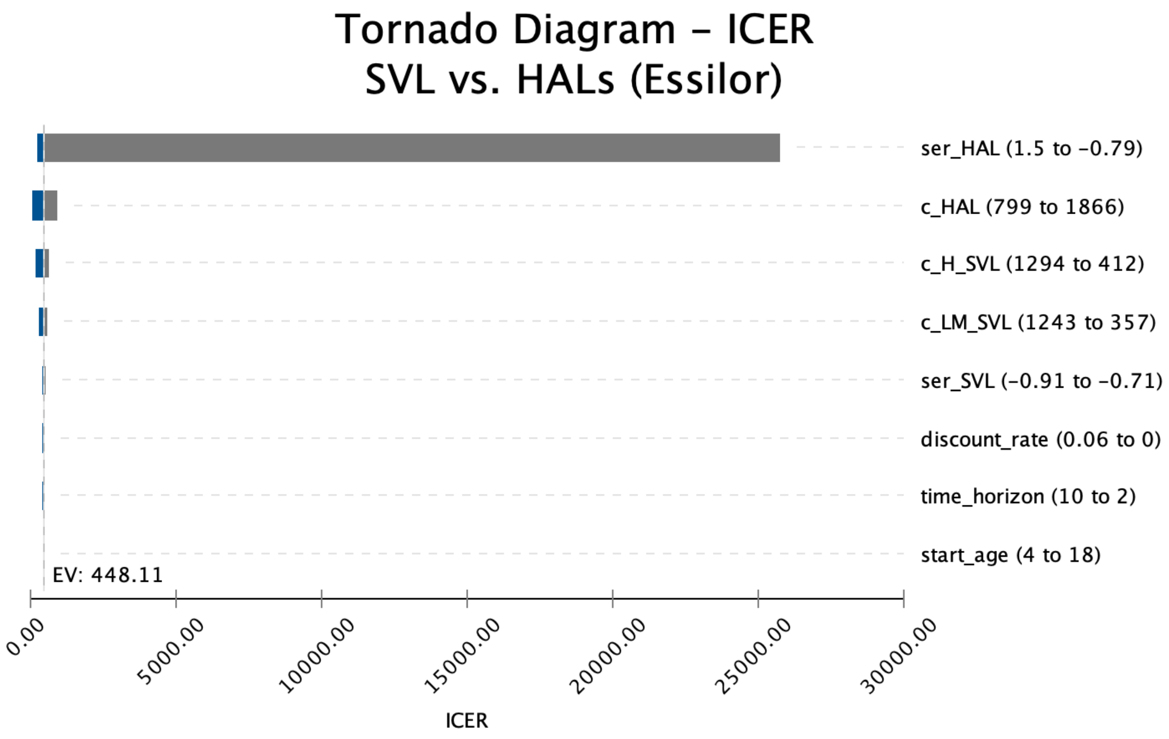

c_HAL = cost of highly aspherical lenslets (HAL)
c_ LM_SVL = cost of single vision lenses for low to moderate myopia
c_H_SVL = cost of single vision lenses for high myopia
ser_HAL = spherical equivalent refraction of HAL
ser_SVL = spherical equivalent refraction of single vision lenses

© 2023 Agyekum S et al. *JAMA Network Open*.

g.

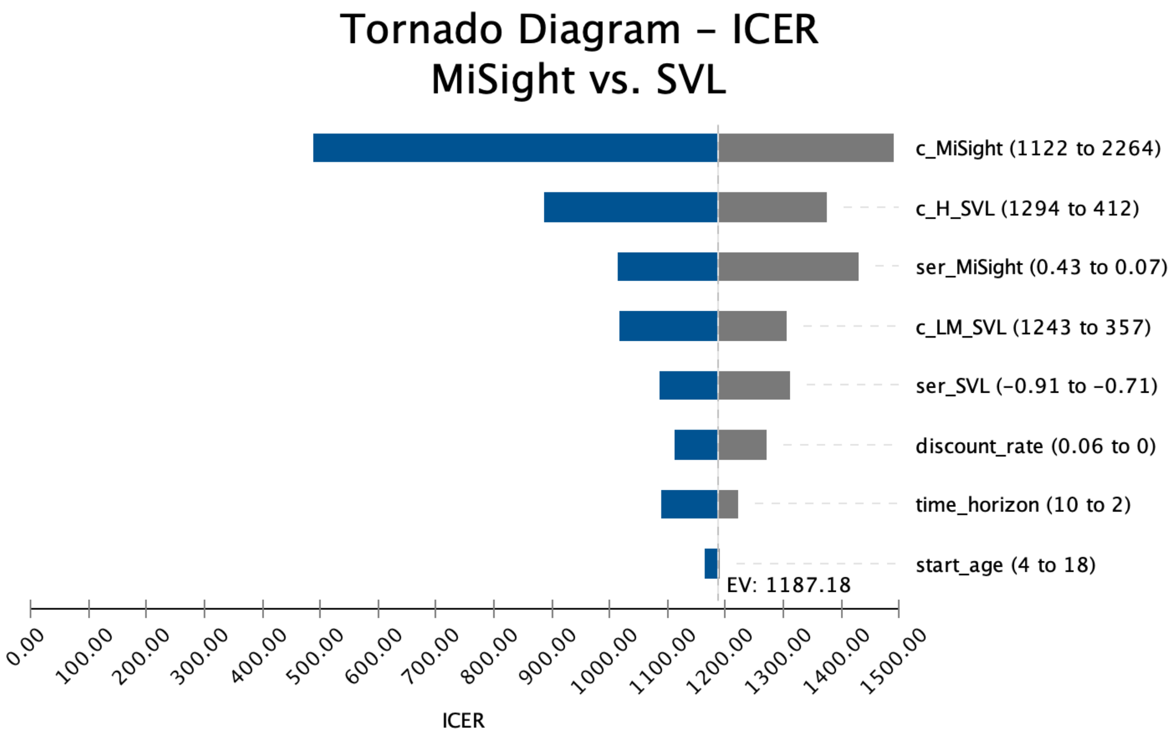

c_MiSight = cost of MiSight lenses
c_ LM_SVL = cost of single vision lenses for low to moderate myopia
c_H_SVL = cost of single vision lenses for high myopia
ser_ MiSight = spherical equivalent refraction of MiSight lenses
ser_SVL = spherical equivalent refraction of single vision lenses

© 2023 Agyekum S et al. *JAMA Network Open*.

h.

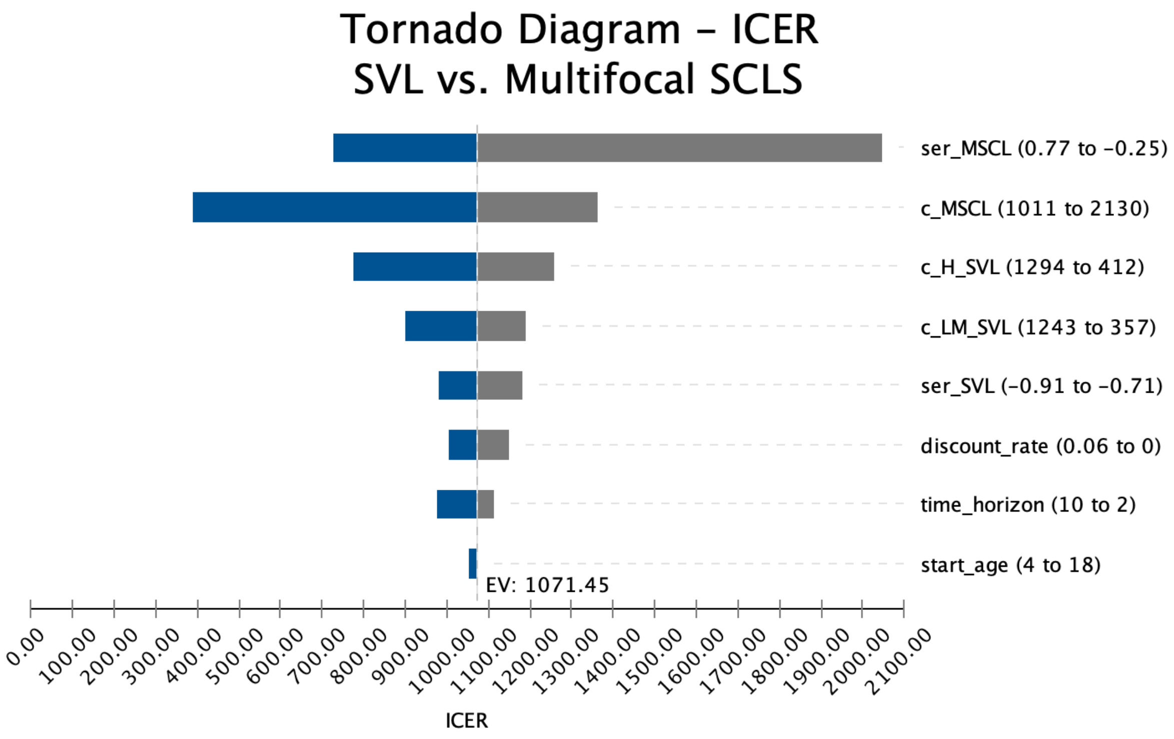

c_MSCL = cost of multifocal soft contact lenses (MSCLs)
c_ LM_SVL = cost of single vision lenses for low to moderate myopia
c_H_SVL = cost of single vision lenses for high myopia
ser_ MSCL = spherical equivalent refraction of MSCLs
ser_SVL = spherical equivalent refraction of single vision lenses

© 2023 Agyekum S et al. *JAMA Network Open*.

i.

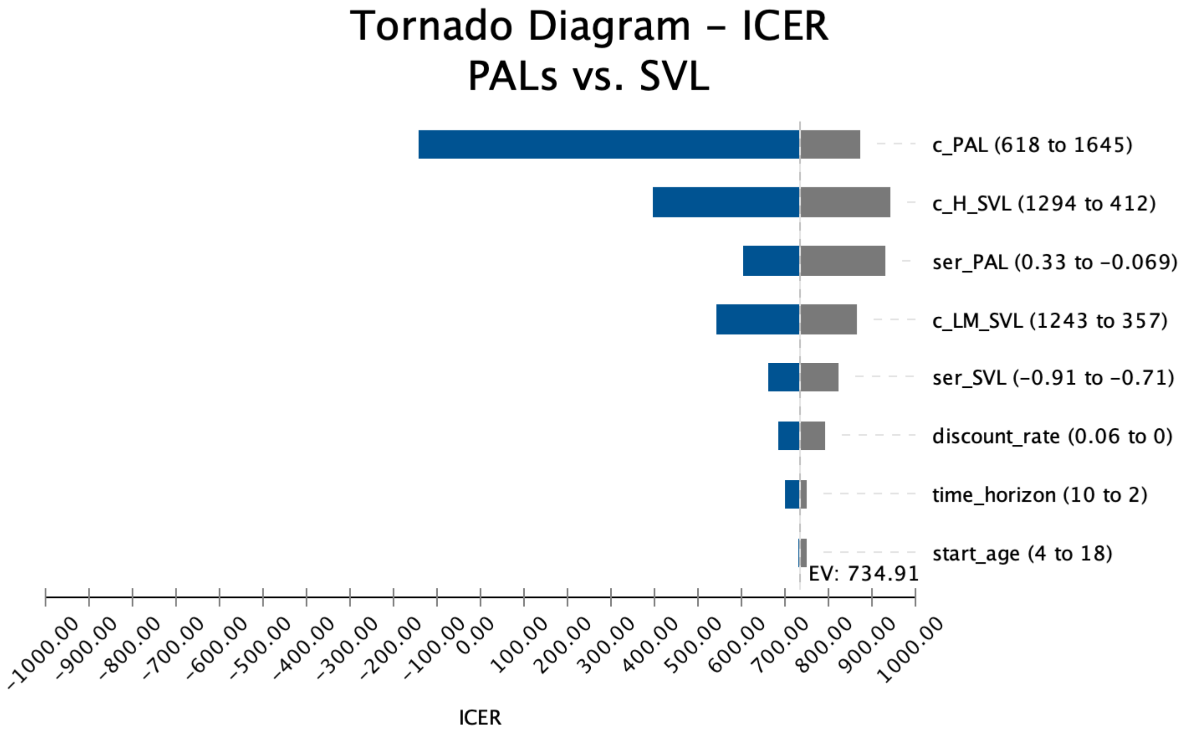

c_PAL = cost of progressive addition lenses (PALs)
c_ LM_SVL = cost of single vision lenses for low to moderate myopia
c_H_SVL = cost of single vision lenses for high myopia
ser_ PAL = spherical equivalent refraction of PALs
ser_SVL = spherical equivalent refraction of single vision lenses

© 2023 Agyekum S et al. *JAMA Network Open*.

j.

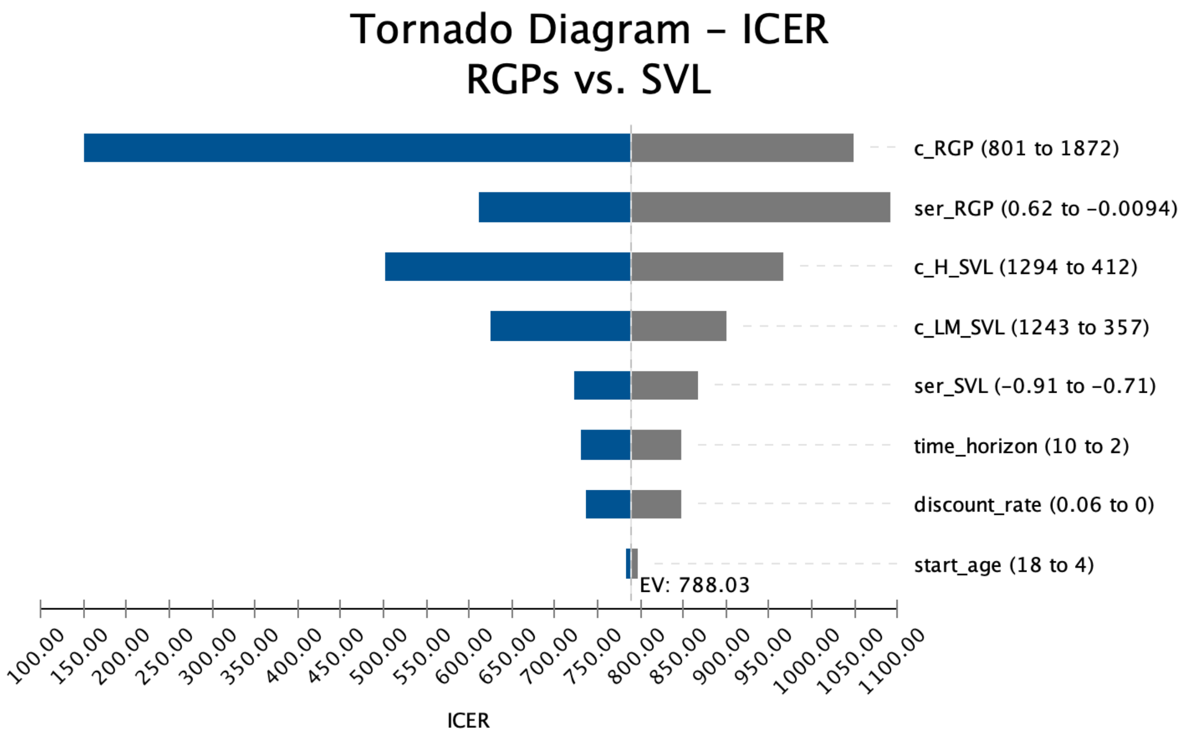

c_RGP = cost of rigid gas permeable (RGP) contact lenses
c_ LM_SVL = cost of single vision lenses for low to moderate myopia
c_H_SVL = cost of single vision lenses for high myopia
ser_ RGP = spherical equivalent refraction of RGP contact lenses
ser_SVL = spherical equivalent refraction of single vision lenses

© 2023 Agyekum S et al. *JAMA Network Open*.

k.

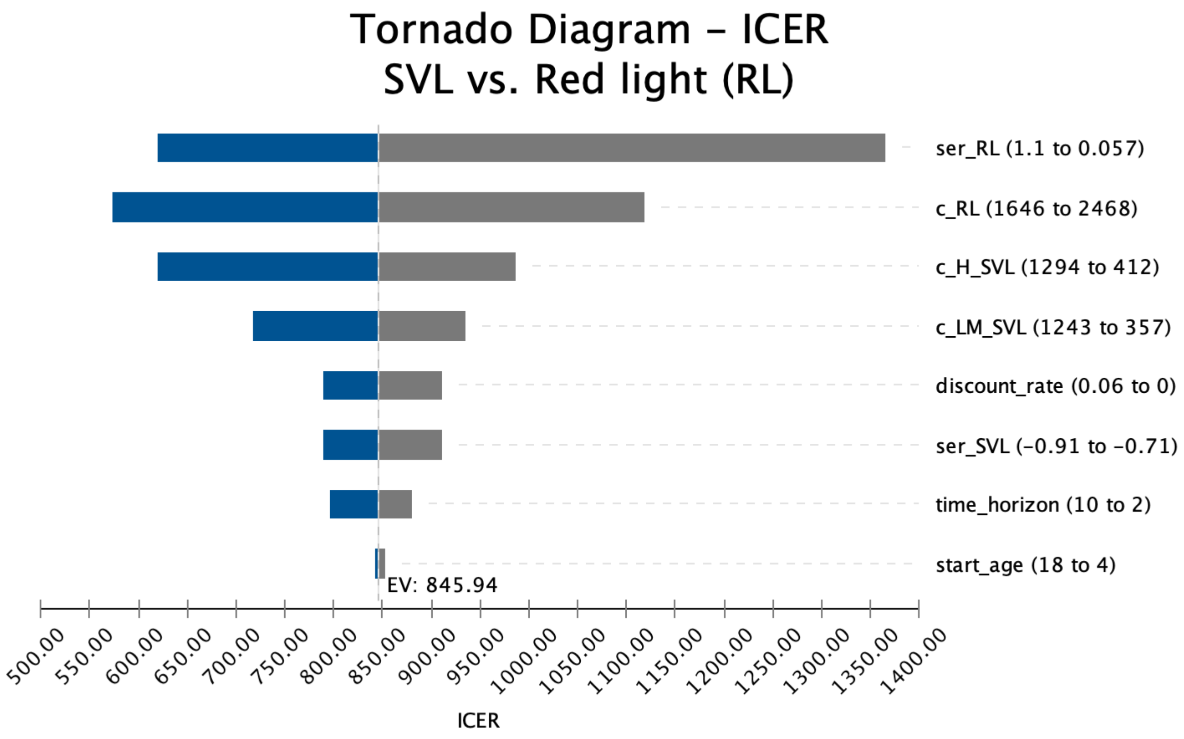

c_RL = cost of red light therapy
c_ LM_SVL = cost of single vision lenses for low to moderate myopia
c_H_SVL = cost of single vision lenses for high myopia
ser_ RL = spherical equivalent refraction of red light therapy
ser_SVL = spherical equivalent refraction of single vision lenses

Tornado diagrams showing the parameters that had the greatest impact on the model**.**
The results show that our model was most sensitive to the costs of the interventions and SER. Increasing cost resulted in less cost-effectiveness.

© 2023 Agyekum S et al. *JAMA Network Open*.

eFigure 3. Cost-Effectiveness Acceptability Curves in Terms of Axial Length

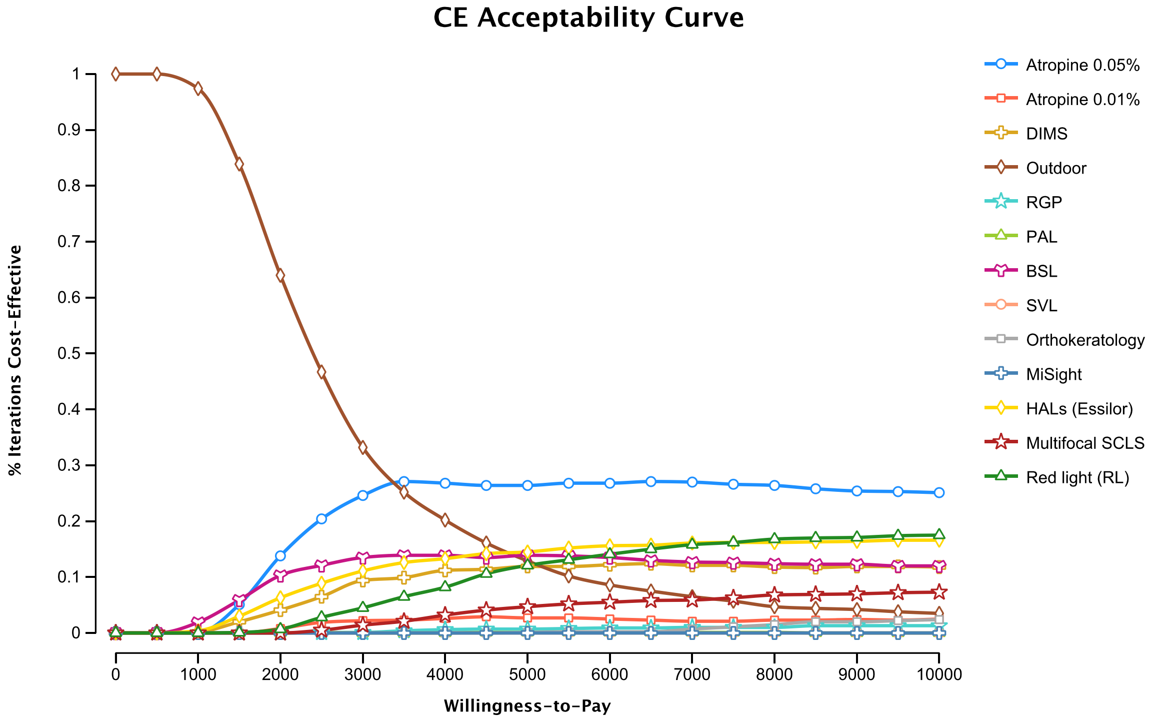

Outdoor activity was the most likely cost-effective intervention at a lower willingness to pay threshold of between 0 to US $3000. Atropine 0.05% was mostly the likely cost-effective intervention above this threshold.

Abbreviations: SVL, single vision lenses; DIMS, defocus incorporated multiple segment spectacles; BSL, bifocal spectacle lenses; PALs, progressive addition spectacle lenses; RGPs, rigid gas-permeable contact lenses; SCLs, soft contact lenses; HALs, highly aspherical lenslets.

© 2023 Agyekum S et al. *JAMA Network Open*.

eReferences

1. Gidwani, R. & Russell, L. B. Estimating Transition Probabilities from Published Evidence: A Tutorial for Decision Modelers. *Pharmacoeconomics* **38**, 1153–1164 (2020).
2. Yam, J. C. *et al.* Low-Concentration Atropine for Myopia Progression (LAMP) Study: A Randomized, Double-Blinded, Placebo-Controlled Trial of 0.05%, 0.025%, and 0.01% Atropine Eye Drops in Myopia Control. *Ophthalmology* **126**, 113–124 (2019).
3. Wu, P. C. C. *et al.* Myopia Prevention and Outdoor Light Intensity in a School-Based Cluster Randomized Trial. *Ophthalmology* **125**, 1239–1250 (2018).
4. Cho, P. & Cheung, S. W. Retardation of myopia in orthokeratology (ROMIO) study: A 2-year randomized clinical trial. *Investig. Ophthalmol. Vis. Sci.* **53**, 7077–7085 (2012).
5. Welton, N. J. & Ades, A. E. Estimation of Markov chain transition probabilities and rates from fully and partially observed data: Uncertainty propagation, evidence synthesis, and model calibration. *Med. Decis. Mak.* **25**, 633–645 (2005).
6. Fulk, G. W., Cyert, L. A. & Parker, D. E. A randomized trial of the effect of single-vision vs. bifocal lenses on myopia progression in children with esophoria. *Optom. Vis. Sci.* **77**, 395–401 (2000).
7. Lam, C. S. Y. *et al.* Defocus incorporated multiple segments (DIMS) spectacle lenses slow myopia progression: A 2-year randomised clinical trial. *Br. J. Ophthalmol.* **104**, 363–368 (2020).
8. Bao, J. *et al.* Spectacle Lenses With Aspherical Lenslets for Myopia Control vs Single-Vision Spectacle Lenses: A Randomized Clinical Trial. *JAMA Ophthalmol.* **140**, 472–478 (2022).
9. Gwiazda, J. E. Progressive-addition lenses versus single-vision lenses for slowing progression of myopia in children with high accommodative lag and near esophoria. *Investig. Ophthalmol. Vis. Sci.* **52**, 2749–2757 (2011).
10. Katz, J. *et al.* A randomized trial of rigid gas permeable contact lenses to reduce progression of children's myopia. *Am. J. Ophthalmol.* **136**, 82–90 (2003).
11. Chamberlain, P. *et al.* A 3-year Randomized Clinical Trial of MiSight Lenses for Myopia Control. *Optom. Vis. Sci.* **96**, (2019).
12. Sankaridurg, P. *et al.* Myopia control with novel central and peripheral plus contact lenses and extended depth of focus contact lenses: 2 year results from a randomised clinical trial. *Ophthalmic Physiol. Opt.* **39**, 294–307 (2019).
13. Jiang, Y. *et al.* Effect of Repeated Low-Level Red-Light Therapy for Myopia Control in Children: A Multicenter Randomized Controlled Trial. *Ophthalmology* **129**, 509–519 (2022).
14. Husereau, D. *et al.* Consolidated Health Economic Evaluation Reporting Standards 2022 (CHEERS 2022) Statement: Updated Reporting Guidance for Health Economic Evaluations. *Clin. Ther.* **44**, 158–168 (2022).

© 2023 Agyekum S et al. *JAMA Network Open*.
