# Supplementary material for: Cost-Effectiveness Analysis of Myopia Progression Interventions in Children
Source: JAMA Netw Open. 2023 Nov 2;6(11):e2340986. doi: 10.1001/jamanetworkopen.2023.40986 (PMC10623196; doi:10.1001/jamanetworkopen.2023.40986)
Supplement: Supplement 2. — Data Sharing Statement [file jamanetwopen-e2340986-s002.pdf]

Data Sharing Statement

Agyekum. Cost-Effectiveness Analysis of Myopia Progression Interventions in Children. *JAMA Netw Open*. Published November 02, 2023. doi:10.1001/jamanetworkopen.2023.40986

Data
Data available: No
